# JUMP AROUND, JUMP AROUND: TRANSPOSABLE ELEMENT ACTIVATION IN NEURODEGENERATIVE TAUOPATHY

**DOI:** 10.1093/geroni/igz038.2178

**Published:** 2019-11-08

**Authors:** Bess E Frost, Wenyan Sun, Hanie Samimi, Habil Zare

**Affiliations:** 1 University of texas health san antonio, San Antonio, Texas, United States; 2 Texas state university, San Marcos, Texas, United States

## Abstract

Transposable elements, or “jumping genes,” constitute ~45% of the human genome. We have identified transposable element activation as a key mediator of neurodegeneration in tauopathies, a group of disorders that are pathologically defined by deposits of tau protein in the brain. Cellular defenses that limit transposable element mobilization include 1) formation of silencing heterochromatin and 2) generation of piwi-interacting RNAs (piRNAs) that clear transposable element transcripts. Using genetic approaches in Drosophila models of tauopathy, we find evidence for a causal relationship between tau-induced heterochromatin decondensation and piRNA depletion, transposable element mobilization, and neurodegeneration. 3TC, an FDA-approved inhibitor of reverse transcriptase, suppresses transposable element mobilization and neuronal death in tau transgenic Drosophila. We detect a significant increase in transcripts of the human endogenous retrovirus class of transposable elements in postmortem human Alzheimer’s disease brains. Our data identify transposable element activation as a conserved, pharmacologically targetable driver of neurodegeneration in tauopathy.

